# Implications of internationalisation of medical education

**DOI:** 10.1186/s12909-023-04630-5

**Published:** 2023-09-07

**Authors:** Marjo Wijnen-Meijer

**Affiliations:** Technical University of Munich, School of Medicine, TUM Medical Education Center, Ismaninger Straße 22, 81675 Munich, Germany

## Abstract

Internationalisation of medical education encompasses the integration of global dimensions and intercultural experiences into medical practices. This process is planned to prepare globally conscious, culturally competent medical workforce that can efficiently address international health challenges. This article describes the impact of internationalisation of medical education on students, teachers and patients.

## Introduction

International health crises, such as SARS, Ebola virus, or COVID-19 pandemic, affected human health, societies and health care provides at different levels. Such crises show the significance of effective global collaboration and communication in education, biomedical research, and patient care. To tackle international health emergencies, culturally competent and highly skilled medical professionals’ leadership, efficient international communication, rapid global health response, and collaborations on medical science and biotechnology research at international level are needed [1]. Therefore, it is crucial for healthcare professionals to comprehend international health and wellness perspectives [2].

The internationalisation of medical education (IoME) plays a key role in equipping healthcare professionals with prerequisite global skills and mindsets to carry out cross-border transformative work in practice [1, 2].

The concept of IoME encompasses systematic, intentional, and evidence-based activities planned to enable students to achieve learning outcomes by participating in high-quality learning opportunities [1]. Thus, IoME comprises specific programmes and procedures that are designed to integrate global dimensions to medical education [1, 3]. To further conceptualise IoME, three major models have been identified by International Higher Education: the “market model,” which focuses on competitiveness of students and academic institutions globally; the “liberal model,” that emphasis intercultural and international cooperation and understanding; and the “social transformation model,” which aims to support equity and social justice in healthcare and links IoME with global health and education domestically and internationally [4, 5].

Additionally, the integration of IoME is possible at curriculum, faculty, and student level — based on the three-dimensional model approach: The traditional model covers a local curriculum, local student, and local teacher; the international medical graduate (IMG) is described by a local curriculum, local teacher, and international student; and the global or translational model comprises an international curriculum, international teacher, and international student [6].

IoME extends beyond reformations in the medical curriculum and can involve a wide range of stakeholders and attributes such as institutions, faculty, students, and government [6].

## Experiences with internationalisation of medical education

IoME has brought numerous experiences such as curriculum expansion, cross-culture exposure, global collaboration and institutional partnership [2]. IoME established on primary principle of internationalisation of the curriculum aims to endorse access of international experiences and education for all medical students abroad and locally. Thus far, it appears that international experience opportunities only exist for a privileged group of medical graduates from prestigious institutions excluding several subgroups of medical students and institutions. This makes IoME socially inequitable [2].

In the past few years, the importance of IoME has been increased significantly as medical institutions aim to produce globally competent and empathic graduates who are capable to practice medicine in miscellaneous cultural settings [3, 7, 8].

International global collaboration and institutional partnerships can also build a huge global medical community. To tackle global health crises, the efficient use of social media and modern technology led by institutional partnerships will be important in near future. Therefore, current technology should be further expanded and incorporated in the medical school curriculum. International institutional partnerships seem to be a mutual practice of IoME but have a great variation in impact and strength [1]. Literature typically reported institutional partnerships among high-income countries (HICs) and low- and middle-come countries (LMICs). Such collaborations can restrict students from acknowledging different healthcare system and collaborative opportunities worldwide, getting to know other HICs and integrating best performances from each setting into their training [1, 9].

### Effects of internationalisation of medical education on teachers

IoME implementation requires multiple players at different levels including medical educators, medical professional, administration, government, students, and faculty [10]. A significant stakeholder for IoME is the faculty — IoME strategies for faculty encompasse teaching, research, and services [1]. There are different ways to implement global dimensions into the curricula of medical schools, e.g., through the placement of a scholar or teacher in an institution abroad for variable durations. The faculty staff may also be a visiting professor or virtual teacher [6]. Additionally, students can utilise internet and e-learning opportunities of one country that are offered and facilitated by faculty staff in another country. For instance, the International Virtual Medical School (IVIMEDS), where schools from sixteen different countries share learning materials [6, 11].

### Effects of internationalisation of medical education on students

Students are primary stakeholders of IoME and may perform various activities in the context of IoME such as attending lectures (“IoME at home”), networking and peer-to-peer connections, gaining intercultural experiences in clinical practices, inbound mobility involving international student employment, and outbound mobility exercises [1].

IoME provides an important platform for students to develop general competencies, such as problem solving, appreciation, collaboration, professionalism, tolerance, empathy, and personal development; leadership skills by participating in extracurricular activities to train themselves in ownership of projects, managerial abilities, and independence; and basic science experience by participation on summer internship programs conducted in basic science laboratories [3].

Institutional partnership also plays an important role in facilitation of peer connections between faculty and students. Advanced technology, like videoconferencing, also connects students from home to international community of students, professionals, or researchers [1].

### Effects of internationalisation of medical education on patients

Finally, IoME can significantly contribute to patient care explicitly or implicitly by facilitating the access to healthcare system, supporting patient-centered care, and promoting culture competence. The IoME comprises for example acquiring knowledge, and comprehending differences in global healthcare education, healthcare delivery services, health laws, heath ethics, and health economics, and establishing global peer networks. IoME further aims to equip prospective physicians with expertise in international competencies, teamwork, and leadership to prepare them best for their future tasks Accordingly, IoME provides knowledge and raises awareness about existing international cultural, economic, social, and ethical disparities in healthcare systems and patients and strengthens effective communication, collaborations, understandings, and feelings of belongingness to a global medical community [1].

### Summary

Nowadays, the IoME can notably contribute to emerging healthcare globalisation and optimise the healthcare worldwide. IoME is playing a key role in integrating international, cross-culture, or global aspects into medical education to promote healthcare quality. This program is particularly designed to enable including researchers, faculty members, students, patients, physicians, institutions, and government to efficiently serve medical community globally.

Articles on the mentioned or other effects of internationalisation on medical education, are welcome for the Collection “Internationalisation of Medical Education.”

## Data Availability

Not applicable.

## Abbreviations

IoME: Internationalization of Medical Education
HICs: High-income countries
LMICs: low-and-middle-come countries
IMG: international medical graduate
IVIMEDS: International Virtual Medical School
